# Metabolic Signaling in a Theoretical Model of the Human Retinal Microcirculation

**DOI:** 10.3390/photonics8100409

**Published:** 2021-09-23

**Authors:** Julia Arciero, Brendan Fry, Amanda Albright, Grace Mattingly, Hannah Scanlon, Mandy Abernathy, Brent Siesky, Alice Verticchio Vercellin, Alon Harris

**Affiliations:** 1 Department of Mathematical Sciences, Indiana University-Purdue University, Indianapolis, IN 46202, USA; 2 Department of Mathematics and Statistics, Metropolitan State University of Denver, Denver, CO 80204, USA; 3 Department of Mathematics and Statistics, Wake Forest University, Winston Salem, NC 27109, USA; 4 Department of Mathematics, Wisconsin Lutheran College, Milwaukee, WI 53226, USA; 5 Icahn School of Medicine at Mt. Sinai, New York, NY 10029, USA

**Keywords:** mathematical model, retina, microcirculation, metabolic signaling, oxygen transport, blood flow, glaucoma, heterogeneous vascular network

## Abstract

Impaired blood flow and oxygenation contribute to many ocular pathologies, including glaucoma. Here, a mathematical model is presented that combines an image-based heterogeneous representation of retinal arterioles with a compartmental description of capillaries and venules. The arteriolar model of the human retina is extrapolated from a previous mouse model based on confocal microscopy images. Every terminal arteriole is connected in series to compartments for capillaries and venules, yielding a hybrid model for predicting blood flow and oxygenation throughout the retinal microcirculation. A metabolic wall signal is calculated in each vessel according to blood and tissue oxygen levels. As expected, a higher average metabolic signal is generated in pathways with a lower average oxygen level. The model also predicts a wide range of metabolic signals dependent on oxygen levels and specific network location. For example, for high oxygen demand, a threefold range in metabolic signal is predicted despite nearly identical PO2 levels. This whole-network approach, including a spatially nonuniform structure, is needed to describe the metabolic status of the retina. This model provides the geometric and hemodynamic framework necessary to predict ocular blood flow regulation and will ultimately facilitate early detection and treatment of ischemic and metabolic disorders of the eye.

## 1. Introduction

Eye disease and associated blindness are highly impactful to both individuals and society as a whole. Despite their obvious importance, the exact cellular mechanisms involved in many ocular pathologies are not well described due to the inaccessibility of deep ocular tissues and the complex physiological interactions between systems. For instance, primary open-angle glaucoma (OAG), a leading cause of irreversible blindness world-wide, is characterized by progressive retinal ganglion cell death and loss of visual field. Reduction of intraocular pressure (IOP) remains the only currently approved treatment in OAG, yet lowering of IOP has not prevented or fully arrested the disease. Although many studies have identified specific aspects of the ocular vasculature to be involved in OAG [1], a clear understanding of mechanistic events during homeostatic hemodynamic regulation and the disease process remains elusive. Previous experimental studies have established relationships between retinal structure and visual function in glaucoma, while others have demonstrated a correlation between impaired blood flow and glaucoma. However, a unified theory of the structural and hemodynamic factors that combine to cause functional visual impairment in glaucoma is missing. Other diseases such as diabetic retinopathy have clear vascular involvement and clinical presentation but lack a robust understanding of prior sequential events and/or individual susceptibility. In both examples, there are significant gaps in knowledge related to the retinal microvasculature, tissue oxygenation, and retinal regulatory capacity during stresses related to disease processes.

To date, mathematical models of the retinal microvasculature and tissue have included only some aspects of the main elements impacting retinal blood flow and oxygenation. The bulk structure of the retinal tissue is multilayered, and the vessel network supplying the tissue is heterogeneous, with wide variations in vessel size, spacing, density, and path length. Models for oxygen diffusion in tissue (e.g., Krogh cylinder model) vary according to simplifying assumptions and network geometry. Previous modeling studies have accounted for blood flow regulation in the retinal microcirculation [2,3]; however, these have assumed a uniform structure to the network and have not included multiple depths of retinal tissue. Studies that have included a more complex network and tissue structure have not accounted for flow regulation [4,5]. A recent theoretical study [6] used mathematical modeling to identify three possible factors that can lead to increased venous oxygen saturation, demonstrating that clinical measures are not sufficient to explain or identify the underlying mechanisms causing them. The multilayer tissue geometry, heterogeneous vascular network structure, flow regulation, and oxygen transport in the retina each contributes in a significant way to the accurate depiction of retinal blood flow; thus, a model that combines all of these factors is greatly needed to make more definite predictions of retinal oxygenation in health and disease conditions.

The current study serves as a necessary first step that will allow for the integration of clinical measures with theoretical models to relate changes in tissue-specific blood flow and oxygenation to visual function and structure in healthy and glaucomatous eyes. In particular, a mathematical model was developed here that can be used to predict oxygen transport and components of blood flow regulation in a heterogeneous description of the retinal microvasculature. The model combined a heterogeneous description of the retinal arterioles with a compartmental model of retinal capillaries and venules. Including a compartmental representation of the downstream microvessels allowed for realistic predictions of PO_2_ downstream of the arterioles. Regulation of blood flow in the microcirculation depended in part upon metabolic signals generated and conducted upstream throughout the entire microvascular network. This model allowed for the prediction of conducted metabolic signals along all nonuniform pathways throughout the network, leading to a spatially heterogeneous distribution of metabolic responses throughout the realistic network of regulating arterioles and, more importantly, providing more accurate predictions of downstream conducted metabolic signals. The implications of the model are highly relevant for glaucoma and other ocular pathologies that involve and potentially alter retinal metabolism and ultimately threaten ganglion cell preservation.

## 2. Materials and Methods

### 2.1. Network Geometry

#### 2.1.1. Arteriolar Network Description

**Figure 1.**
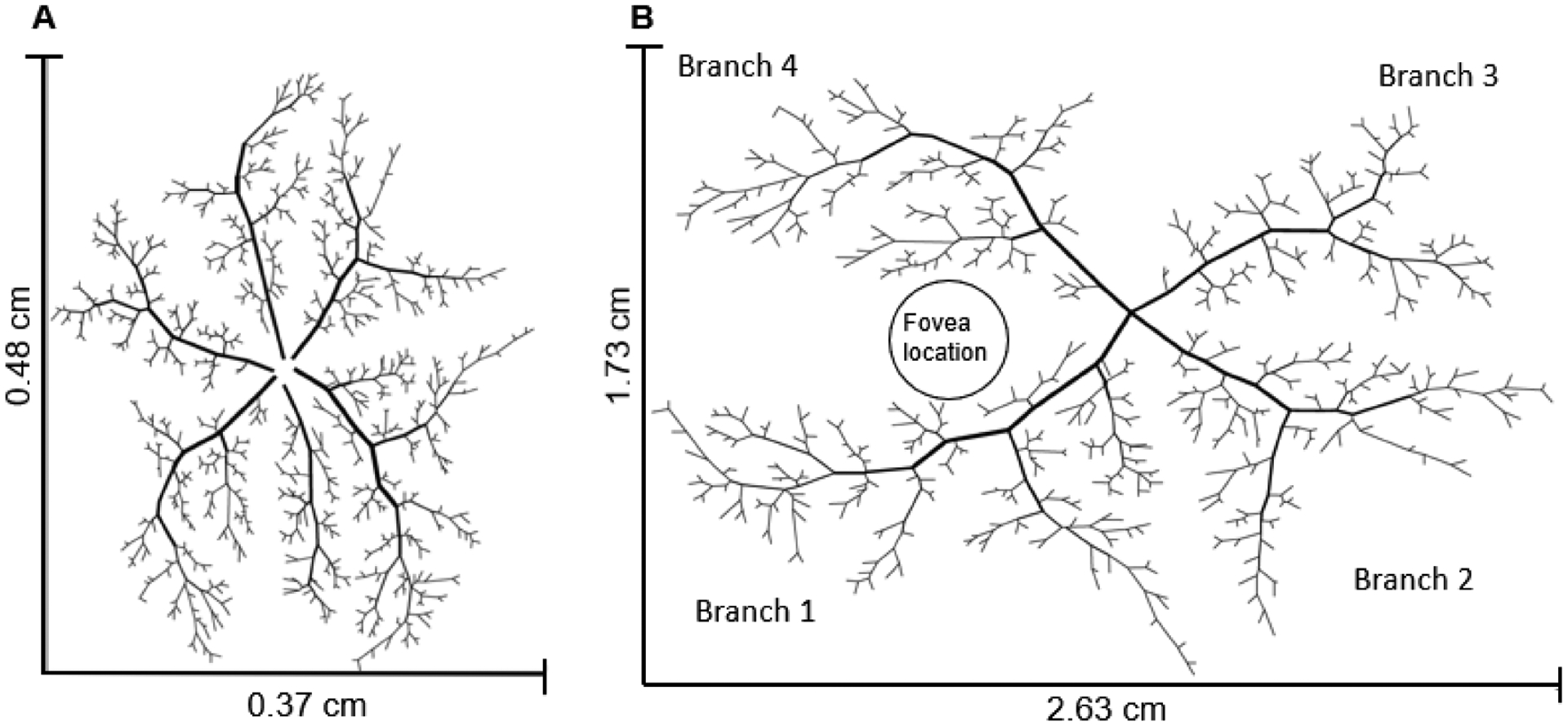
(**A**) Heterogeneous mouse arteriolar network obtained using position, length, and diameter data from [8,9], as described in [7]. (**B**) Heterogeneous human arteriolar network developed by modifying the mouse model in panel (**A**) in the following ways: reducing the number of main branches from six to four, rotating the four main branches according to oximetry images, and increasing vessel diameters and lengths by a scaling factor of 3.6 and 5.9, respectively.

In this study, a theoretical model of human retinal arterioles was extrapolated from a previous model of the mouse retina [7]. In [7], detailed mappings of the positions, lengths, and diameters of the arteriolar network in the mouse retina (Figure 1A) were obtained from confocal microscopy images [8,9]. Since such detailed mappings have not been obtained from a human retina, human oximetry data were used to translate the murine arteriolar network to a human network by adapting three main components: (i) the number of main arterial and venous branches and the angles between them, (ii) vessel diameters, and (iii) vessel lengths. First, two of the six arterial branches in the mouse retinal network were eliminated; the remaining four were repositioned to superior/inferior temporal/nasal positions according to angles calculated from oximetry images and the position of the fovea. Next, based on oximetry biomarkers from human retinal arterioles [10], a scaling factor of 3.6 was used to convert murine vessel diameters to human values. Last, the distance from the CRA to the fovea in the human retina (approximately 4.5 mm) was used to determine a 5.9 scaling factor between mouse and human vessel lengths [11,12]. Importantly, the heterogeneity of the network branching structure was assumed to be similar between species since several studies have indicated that the morphology of the human and murine vascular networks is strikingly similar [13,14]. The resulting human arteriolar network is shown in Figure 1B. To validate this mouse-to-man conversion process, it is noted that an assumed pressure drop of 16 mmHg along the human arteriolar network [2] corresponds to a total flow of 36,670 nL/min to the human retinal microcirculation, which is consistent with the flows measured in human retina [15,16].

#### 2.1.2. Capillary and Venular Network Description

Although the model defined in [7] included a heterogeneous description of the capillaries and venules, the current study represented retinal capillaries (C), small venules (SV), and large venules (LV) using a series of compartments (as in [2,17]) in an effort to obtain insights into flow and oxygenation throughout the entire retina without requiring intense computational power. Each vessel compartment was assumed to contain a set of identical, parallel-arranged segments that experience identical hemodynamic and metabolic conditions. This compartmental model was used to evaluate flow and oxygenation of the retinal microcirculation downstream of the heterogeneous arteriolar network.

#### 2.1.3. Hybrid Model Description

**Figure 2.**
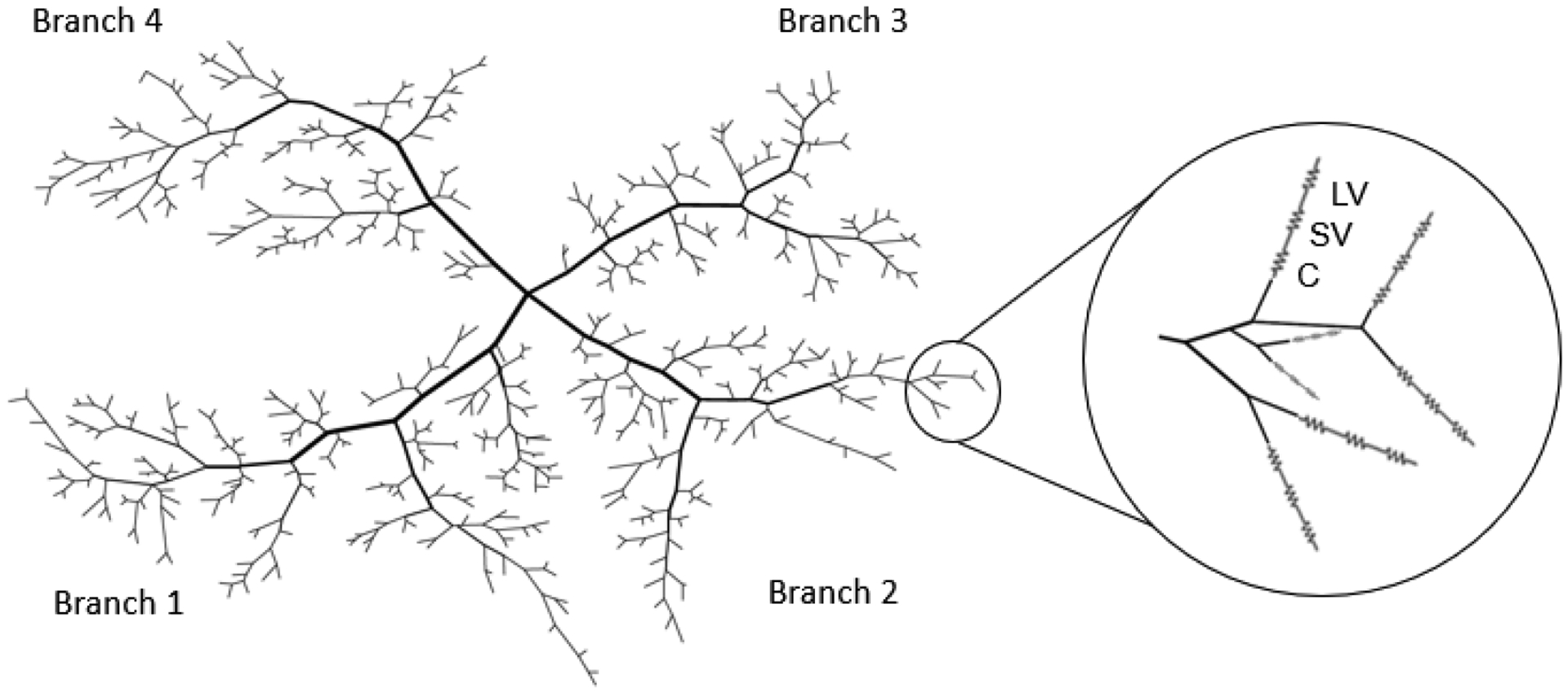
Schematic representation of the hybrid model. The circle shows an enlarged portion of the network where a series of compartments for the capillaries (C), small venules (SV), and large venules (LV) are attached to each terminal arteriole in the heterogenous model.

The theoretical model of the human retinal microcirculation introduced in this study was defined as a hybrid model that combines the heterogeneous model of retinal arterioles described in Section 2.1.1 with a compartmental representation of capillaries and venules described in Section 2.1.2. More specifically, vessel compartments corresponding to capillaries, small venules, and large venules were connected in series to each terminal arteriole in the heterogeneous model. This model expanded previous work [2] by capturing the full heterogeneity of the arteriolar network structure. Since arterioles are the primary microvessels capable of actively regulating flow, this hybrid model allowed for spatial predictions of oxygen distribution and blood flow. The heterogeneity of the arteriolar network was preserved in the downstream compartments by requiring that the inflow oxygen content to the capillary compartment be calculated as a weighted sum of the hematocrit in each of the outflowing arterioles. The hybrid model is depicted in Figure 2.

### 2.2. Blood Flow

The arteriolar network of the hybrid model was represented as a large, directed graph, whereby each vessel with a particular diameter and length was represented by an edge and each junction was represented by a node. Pressure-driven flow through each segment in the network was modeled using Poiseuille’s Law:

(1) $$Q=\frac{\pi \Delta PD^{4}}{128\;\mu \text{L}}$$

where *Q* is the volumetric blood flow rate in an individual vessel segment, Δ*P* is the pressure drop along the vessel, *D* is the diameter of the blood vessel, *L* is the vessel length, and *μ* is the apparent viscosity, which was assumed dependent on the diameter and hematocrit of the blood vessel based on the diameter-dependent relationship previously established by Pries et al. [18].

Conservation of mass was imposed at every junction in the network, which allows for the flow rate, hematocrit, and apparent viscosity to be calculated in each arteriole using an iterative scheme [19], described in detail in [7]. Initial pressure and flow conditions for the capillary compartment were obtained from the predictions in the terminal arterioles of the heterogeneous arteriolar network. Poiseuille’s Law and conservation of mass were then used to compute flows in the downstream compartmental network.

### 2.3. Oxygen Transport

The governing equation for steady-state diffusion with oxygen consumption in the tissue was given by

(2) $$D_{\text{diff}}\alpha \nabla P_{O2}=M\left(P_{O2}\right)$$

where *P*_*O*2_ is the tissue partial pressure of oxygen, *M*(*P*_*O*2_) is the tissue oxygen consumption rate, and *D*_*diff*_ and *α* are the diffusivity and solubility of oxygen in the tissue, respectively. Here, *D*_*diff*_*α* = 6 × 10^−10^ cm^3^ O_2_/cm/s/mmHg [20,21]. The consumption rate in the tissue region was assumed to depend on *P*_*O*2_ following Michaelis–Menten oxygen utilization kinetics, so that

(3) $$M\left(P_{O2}\right)=M_{0}\frac{P_{O2}}{P_{0}+P_{O2}}$$

where *M*_0_ is the tissue oxygen demand, and *P*_0_ is the Michaelis constant at which the consumption rate is half maximal (taken here to be 10 mmHg [22]).

In the spatially heterogeneous arteriolar network, a Green’s function method [23,24] was used to solve Equation (2), where the vessels were modeled as discrete oxygen sources, and the tissue points were represented as oxygen sinks [7,23–25]. The resulting *P*_*O*2_ at a tissue point was then calculated as the superposition of the oxygen fields (Green’s functions) produced by each of the surrounding sources and sinks. This method accounted for diffusive interactions between all vessels and tissue points in the network and was computationally efficient, as it reduced the problem to solving for the strengths of the sources and sinks.

By conservation of mass, in each arteriole,

(4) $$\frac{df\left(P_{b}\right)}{ds}=-q(s)$$

where *f*(*P*_*b*_) = *Q*(*H*_*D*_*C*_0_*S*(*P*_*b*_) + *α*_*b*_*P*_*b*_) is the rate of convective oxygen transport along a vessel segment, *Q* is the blood flow rate, *H*_*D*_ is the discharge hematocrit, *C*_0_ is the concentration of hemoglobin-bound oxygen in a fully saturated red blood cell, *P*_*b*_ is the blood PO_2_, *s* is the distance along the vessel segment, *q*(*s*) is the rate of diffusive oxygen efflux per unit vessel length, and *S*(*P*_*b*_) is the oxyhemoglobin saturation, which was assumed to be a function of the blood *P*_*O*2_ via a Hill equation:

(5) $$S\left(P_{b}\right)=\frac{P_{b}^{n}}{P_{b}^{n}+P_{50}^{n}}$$

where *P*_*5*0_ is the vessel *P*_*O*2_ at which hemoglobin is half-saturated, and *n* is the Hill coefficient. Here, *P*_50_ = 26 mmHg and *n* = 2.7 (based on [26]).

Similar to Equation (4), by conservation of mass, the rate of change in oxygen flux in the capillary and venous compartments was given by

(6) $$\frac{d\left(Q_{j}H_{D}C_{0}S\left(P_{b}(s)\right)\right)}{ds}=-q(s)$$

where index *j* denotes compartment, and *s* is distance along the compartment.

In the capillary compartments, oxygen consumption was calculated using a Krogh cylinder model [27], in which each capillary was assumed to provide oxygen via diffusion to a surrounding tissue cylinder according to:

(7) $$D_{\text{diff}}\alpha \left[\frac{1}{r}\frac{d}{dr}\left(r\frac{dP_{O2}(s,r)}{dr}\right)\right]=M\left(P_{O2}\right)$$

where *r* is the radial distance within the tissue cylinder. The tissue oxygen consumption per vessel length was computed as:

(8) $$\int_{r_{v}}^{r_{t}}M\left(P_{O2}\right)2\pi r\;dr$$

where *r*_*t*_ denotes the radius of the tissue and *r*_*v*_ denotes the radius of the vessel (capillary). The width of tissue surrounding each capillary was defined as *d* = *r*_*t*_ − *r*_*v*_. Since oxygen is exchanged primarily in the arterioles and capillaries, oxygen exchange in the venular compartments was neglected (and hence tissue width in the venules was set to zero). Assuming a fixed capillary density of *N* = 50,000/cm^2^, the value for tissue depth *d* = 22 μm was determined by solving Equation (9):

(9) $$N=\frac{\sum_{i}n_{C,i}L_{C,i}}{A_{VOL}+\sum_{i}\left[n_{C,i}L_{C,i}\pi {\left(r_{C,i}+d\right)}^{2}+n_{SV,i}L_{SV,i}\pi r_{SV,i}^{2}+n_{LV,i}L_{LV,i}\pi r_{LV,i}^{2}\right]}$$

where *A*_*VOL*_ = 0.0025 cm^3^ is the total volume of the arteriolar network vessels and tissues, and index *i* denotes each vascular pathway. A fourth-order BVP solver in MATLAB was used to calculate the partial pressure of oxygen in the radial direction while iterating down the vessel segments. If PO_2_ decreased to zero, all subsequent values of PO_2_ along the capillary were also set to zero.

### 2.4. Metabolic Signal Calculation

Regulation of blood flow in the microcirculation depends, in part, upon metabolic signals generated and conducted upstream throughout the entire microvascular network. The model developed in this study allowed for the prediction of conducted metabolic signals along all vascular pathways; this algorithm for determining the conducted metabolic signal will be utilized in future work to predict flow regulation within the hybrid model.

The metabolic response in a given vessel, *S*_*meta*_, was defined as the average value of the metabolic wall signal generated along the length of the vessel [25]. This signal was composed of two exponentially decaying terms: one from the downstream outflow node of the vessel (*S*_*meta,out*_) that was decayed upstream along the length of the vessel, and one from the local signal rate (*S*_*loc*_) generated at every point along the vessel and decayed upstream along the remaining length of the vessel. The local metabolic signal, *S*_*loc*_, generated at each point along the vessel was inversely related to the local vessel *P*_*O*2_:

(10) $$S_{loc}=\frac{P_{0}}{P_{0}+P_{O2}}$$

Thus, in a particular vessel, the metabolic response was given by

(11) $$S_{\text{meta}\;}=\frac{1}{L}\int_{0}^{L}S_{\text{meta},out}e^{-x/L_{0}}dx+\frac{1}{L}\int_{0}^{L}\left[\int_{0}^{x}S_{loc}e^{-(x-y)/L_{0}}dy\right]dx$$

where *L*_0_ is the exponential decay constant, assumed here to be 1 cm, as in [2].

### 2.5. Control State for the Hybrid Model

A control (or reference) state was established in this model to represent baseline geometric and hemodynamic conditions corresponding to a healthy human retina. An incoming pressure (P_a_) of 40 mmHg was assumed at the beginning of the arteriolar network (i.e., the downstream end of the central retinal artery), and an overall pressure drop of 16 mmHg was assumed across the arterioles (consistent with [2]).

Capillary diameter was assumed to be *D*_*C*_ = 6 microns, and shear stress (*τ*) in the capillaries was assumed to be *τ*_*C*_ = 15 dyn/cm^2^. Viscosity in the capillaries (*μ*_*C*_) was given as a function of capillary diameter obtained from an empirical fit to experimental data [18]. Given these values, flow in a capillary was calculated as $Q_{C}=\frac{\pi \tau_{C}D_{C}^{3}}{32\mu_{C}}$.

A loose symmetry assumption was implemented in this study to define geometric components of the venous compartments; the symmetry assumptions (based on those outlined in [2]) required the heterogeneous arteriolar network to be classified in terms of large arterioles (LA) and small arterioles (SA) so that analogous definitions between arterioles and venules could be established. In the heterogeneous arteriolar network, terminal arterioles (i.e., the final small arteriolar segments that connect directly to a capillary compartment) were defined as small arterioles. The entire arteriolar pathway upstream of the terminal arteriole was classified as a large arteriole. Diameters and lengths of all arterioles in the hybrid model were obtained from a scaling of the confocal microscopy images of the murine retina (Section 2.1.1). Since the large arteriole classification contains vessels of many different diameters, *D*_*LA*_ was defined as the diameter of the first (i.e., most upstream) vessel for a particular pathway; the diameter of the SA (*D*_*SA*_) was the diameter of the terminal arteriole (all diameters of terminal arterioles were the same). Flow and pressure drop in the LA and SA were obtained from the heterogeneous model calculations. Specifically, in any given pathway, the flow in the LA (*Q*_*LA*_) was taken as the incoming flow to the first vessel (main branch) of that pathway, and flow in the SA (*Q*_*SA*_) was taken as the flow calculated in the terminal arteriole, which also corresponded to the flow that would enter the downstream compartments. The pressure drop in the LA (Δ*P*_*LA*_) was calculated as the entire pressure drop from the incoming point of the pathway to the upstream end of the terminal arteriole. The pressure drop along the SA (Δ*P*_*SA*_) was the pressure drop in the terminal arteriole. The first symmetry assumption required that the number of vessels (*n*) in corresponding compartments be equal (as in [17]). That is, n_SA_ = n_SV_ and n_LA_ = n_LV_. Since all pathways contain only a single terminal arteriole, n_SA_ = n_SV_ = 1. Conservation of flow along a pathway required that n_LA_Q_LA_ = n_SA_Q_SA_ = n_C_Q_C_ = n_SV_Q_SV_ = n_LV_Q_LV_. As a result, n_LA_ = Q_SA_/Q_LA_ = n_LV_ and n_C_ = Q_SA_/Q_C_.

Wall shear rates (ω) in the retinal microcirculation were given as a function of arterial and venous diameter in [28]. In the present study, wall shear rates were interpolated from the values provided in [28] for capillary, small (terminal) arteriole, and large arteriole diameters. If the large arteriole diameter was outside the range of interpolation, the maximum possible interpolated value was used, which was reasonable, since the wall shear rates were nearly constant for large diameter values. Control state diameter values for the small and large venule from [2] (i.e., *D*_*SV*_ = 69 μm and *D*_*LV*_ = 140 μm) were used here to interpolate for the wall shear rate in the SV and LV compartments. Upon obtaining the wall shear rate for all vessel types, symmetry assumptions and flow conservation were used to calculate venular diameters used in the hybrid model: $D_{SV}=D_{SA}{\left(\frac{\omega_{SA}}{\omega_{SV}}\right)}^{1/3}$ and $D_{LV}=D_{LA}{\left(\frac{\omega_{LA}}{\omega_{LV}}\right)}^{1/3}$. With all control state diameter values defined, viscosity in all vessels was obtained from an empirical relationship [18]. Then, wall shear stress in all compartments was calculated according to *ω* = *τ*/*μ*.

Another symmetry assumption dictated that the lengths of corresponding vessel compartments were equal, i.e., L_SA_ = L_SV_ and L_LA_ = L_LV_. Pressure drops in the small and large venule compartments were obtained using Poiseuille’s Law (Equation (1)). Since a total pressure drop across the entire microcirculation is assumed to be P_a_—IOP, where IOP is the intraocular pressure and was assumed to be 15 mmHg, the remaining pressure drop in the capillary compartment was calculated. Then, the length of the capillary compartment was obtained using Poiseuille’s Law. Table 1 provides the diameter, wall shear stress, pressure drop, number of segments, length, viscosity, and flow averaged over all pathways in the control state for the C, SV, and LV compartments.

### 2.6. Model Algorithm and Simulations

**Figure 3.**
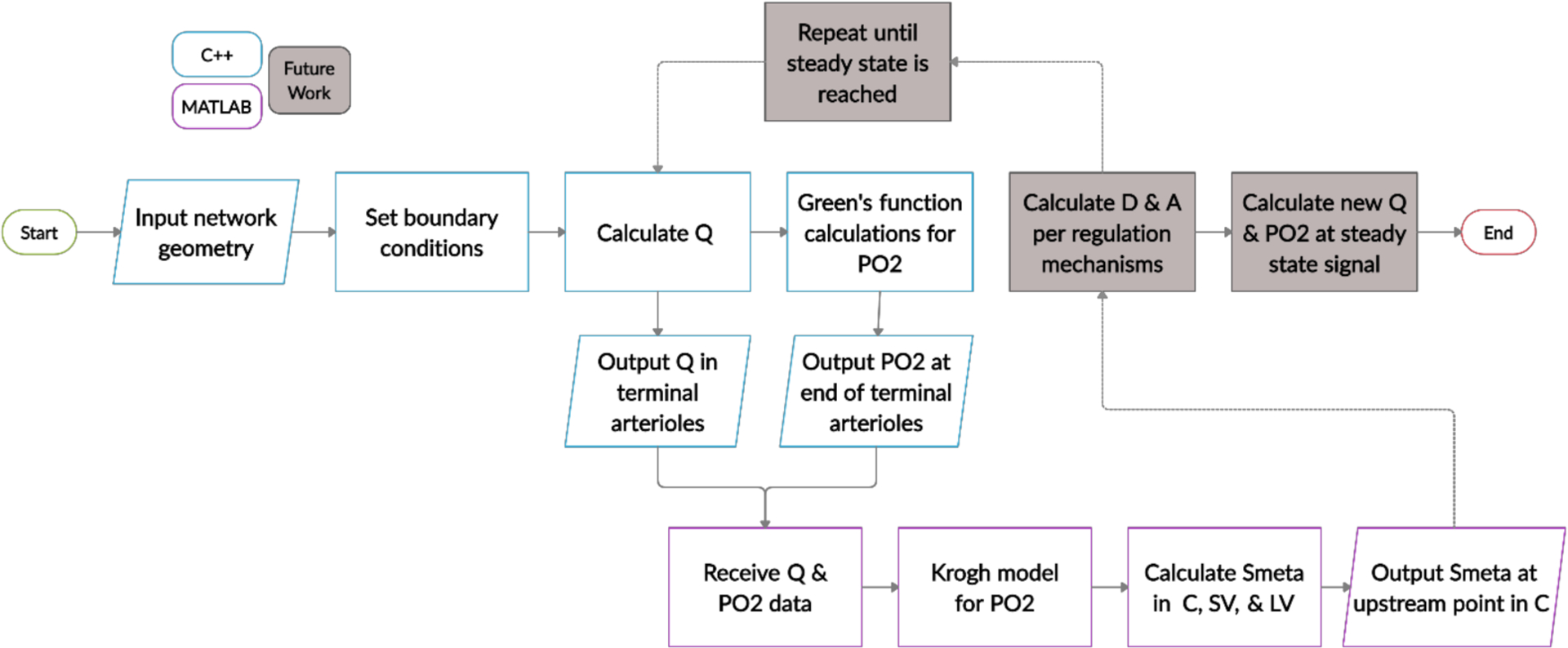
Flowchart of the hybrid model programmed in C++ (blue) and MATLAB (purple). The heterogeneous arteriolar model is programmed in C++ and the compartmental capillary and venular model is in MATLAB. The two programming languages will be dynamically linked to exchange information repeatedly until a steady state of blood flow and diameter activation is achieved (future work, shaded gray).

The heterogenous arteriolar network was programmed using C++, and the compartmental model was programmed in MATLAB. Figure 3 summarizes the numerical procedure implemented in this study (as described in detail in Sections 2.1–2.4); computations using C++ are labeled in blue, and those using MATLAB are labeled in purple. First, as described in Section 2.1.1, the position and diameter of every arteriole were defined. Then, boundary conditions for the heterogeneous network were defined. Here, the incoming arterial pressure was set to 40 mmHg, and the outgoing terminal arterial pressure was set to 24 mmHg, yielding a fixed pressure drop of 16 mmHg along the arterioles, consistent with [2]. The arterioles were assumed to be well oxygenated with an inflow PO_2_ of 84.4 mmHg and saturation of 0.96. Blood flow was calculated within each arteriole, and the Green’s function method was implemented to calculate PO_2_ in tissue points and all arterioles. Values of blood flow, PO_2_, diameter, vessel length, and pressure drop in the terminal arterioles were then sent to MATLAB for use in the compartmental model. In the capillaries, a Krogh cylinder model was used to calculate PO_2_ along the vessel compartment. Using these PO_2_ values, the wall-derived metabolic signal was calculated in the capillaries and venules (see Section 2.4), assuming zero signal at the downstream end of the large venule compartment. The value of the signal at the upstream end of the capillaries was transmitted back to C++ so that the metabolic signal could be calculated throughout the entire arteriolar network. Blood flow, oxygenation, and metabolic signals were predicted as oxygen demand was varied between *M*_0_ = 1 and 4 cm^3^ O_2_/100 cm^3^/min. Although the current study ends with the calculation of this signal, ultimately, the metabolic signal will be used to simulate flow regulation using a previously established vessel wall mechanics model [2,17,25] to obtain more accurate predictions of diameter changes as pressure or metabolic demand are varied. The two programming languages will be dynamically linked to exchange data until a steady state is reached, at which point flows and oxygenation levels will be obtained for the hybrid model (future work, shaded in Figure 3).

## 3. Results

**Figure 4.**
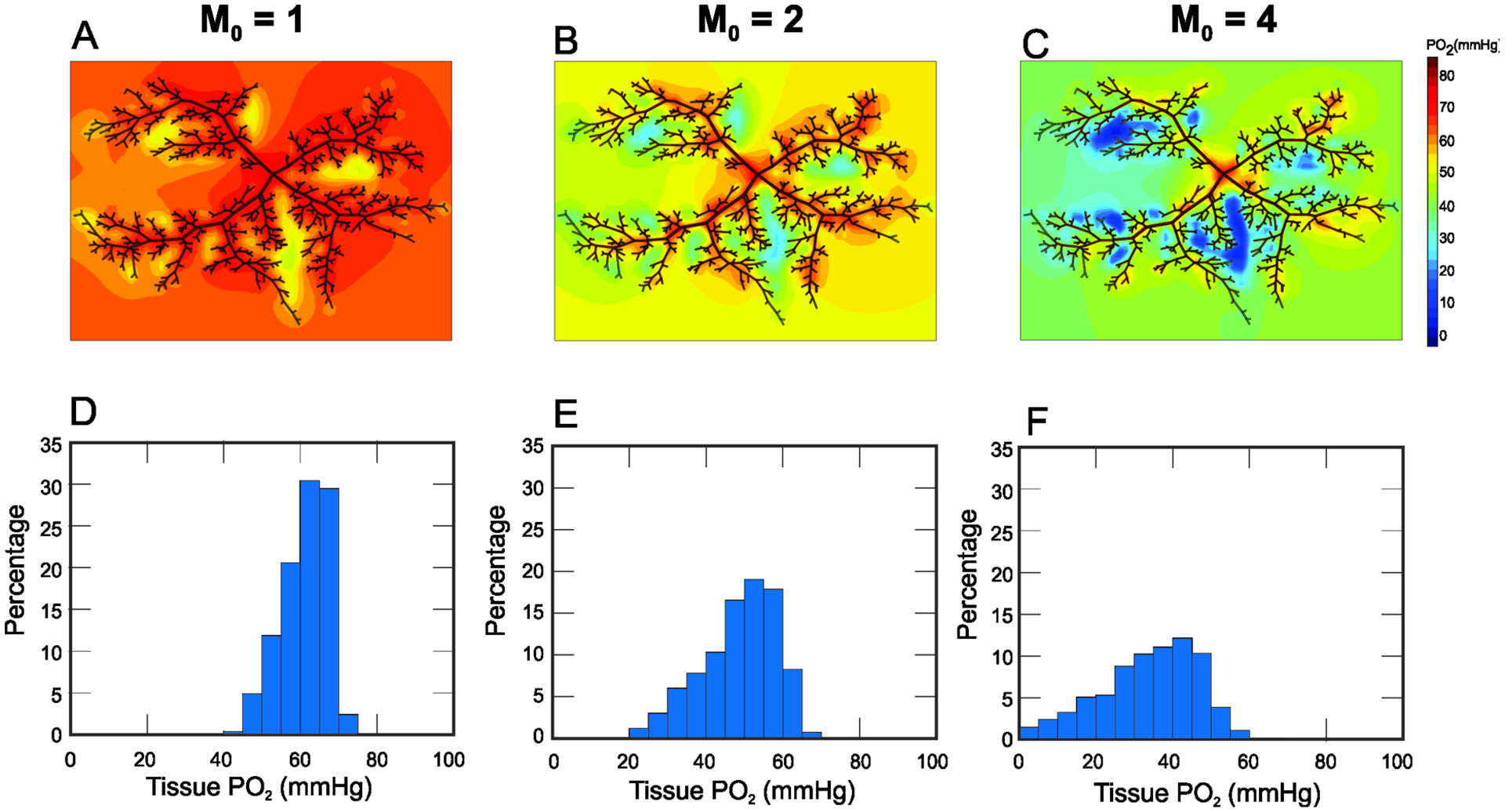
Contour plots (panels (**A**–**C**)) and histograms (panels (**D**–**F**)) of tissue PO_2_ in the arteriolar network for varying levels of oxygen demand (*M*_0_) prior to the calculation of the conducted metabolic response. Three levels of oxygen demand were simulated: low (*M*_0_ = 1 cm^3^ O_2_/100 cm^3^/min, panels (**A**,**D**)), moderate (*M*_0_ = 2 cm^3^ O_2_/100 cm^3^/min, panels (**B**,**E**)), and high (*M*_0_ = 4 cm^3^ O_2_/100 cm^3^/min, panels (**C**,**F**)). As *M*_0_ is increased, a nonuniform decrease in tissue PO_2_ was predicted.

Figure 4 shows the model predicted levels of tissue PO_2_ in the arterioles for three values of oxygen demand. As oxygen demand (*M*_0_) is increased from 1 to 4 cm^3^ O_2_/100 cm^3^/min, there is a nonuniform reduction in PO_2_ throughout the tissue indicated by the blue, green, yellow, and red regions in the contour plots shown in Figure 4A–C. In addition, multiple regions are consistently predicted to exhibit low PO_2_ at each oxygen demand level. Such spatial variance in oxygen distribution could not be predicted without utilizing this heterogeneous description of the arterioles. The histograms in Figure 4D–F quantify the distribution of PO_2_ in the retinal tissue and indicate an increase in the spread of PO_2_ values as oxygen demand is increased.

**Figure 5.**
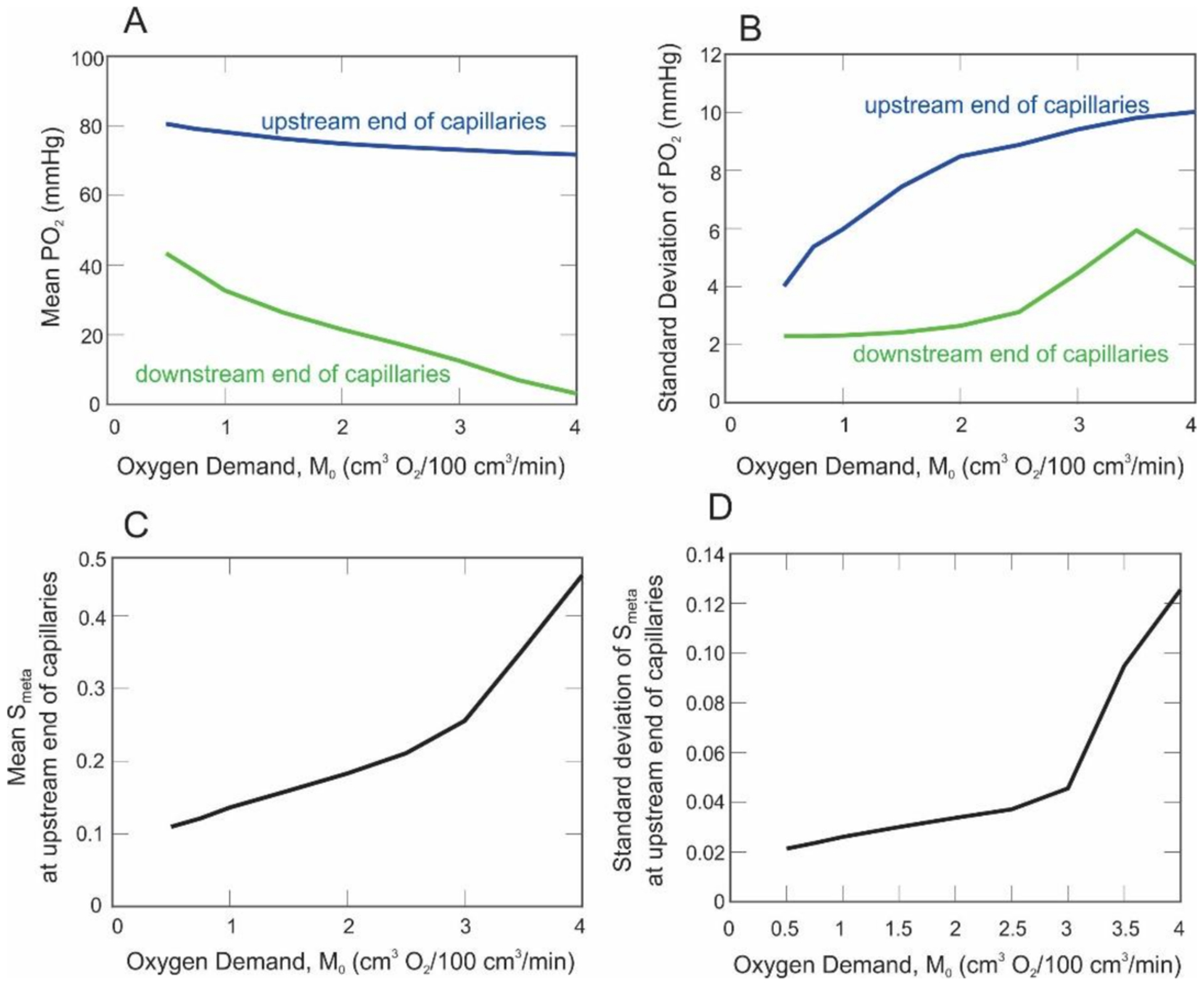
Mean PO_2_ (panel (**A**)) and standard deviation of PO_2_ (panel (**B**)) at the upstream (blue) and downstream (green) end of the capillary compartment as oxygen demand is varied from *M*_0_ = 0.5 to 4 O_2_/100 cm^3^/min. Mean (panel (**C**)) and standard deviation (panel (**D**)) of the metabolic signal (*S*_*meta*_) calculated at the upstream end of the capillaries as oxygen demand is varied.

Figure 5A,B shows the mean and standard deviation, respectively, of PO_2_ at the upstream (blue curve) and downstream (green curve) end of the capillary compartment as oxygen demand increases. As expected, both curves in Figure 5A decrease monotonically with oxygen demand. Since the majority of oxygen extraction occurs in capillaries, the PO_2_ at the upstream end of the capillaries remains relatively high and decreases slightly with increased oxygen demand. The vertical distance between the blue and green curves indicates that oxygen extraction increases in the capillaries as oxygen demand is increased. Unlike the predicted mean PO_2_ values, the standard deviation in PO_2_ is predicted to increase in almost all cases when *M*_0_ is increased from 0.5 to 4 cm^3^ O_2_/100 cm^3^/min (Figure 5B). The decrease in the standard deviation of PO_2_ at high oxygen demand resulted from PO_2_ values equal to zero in multiple vascular pathways.

**Figure 6.**
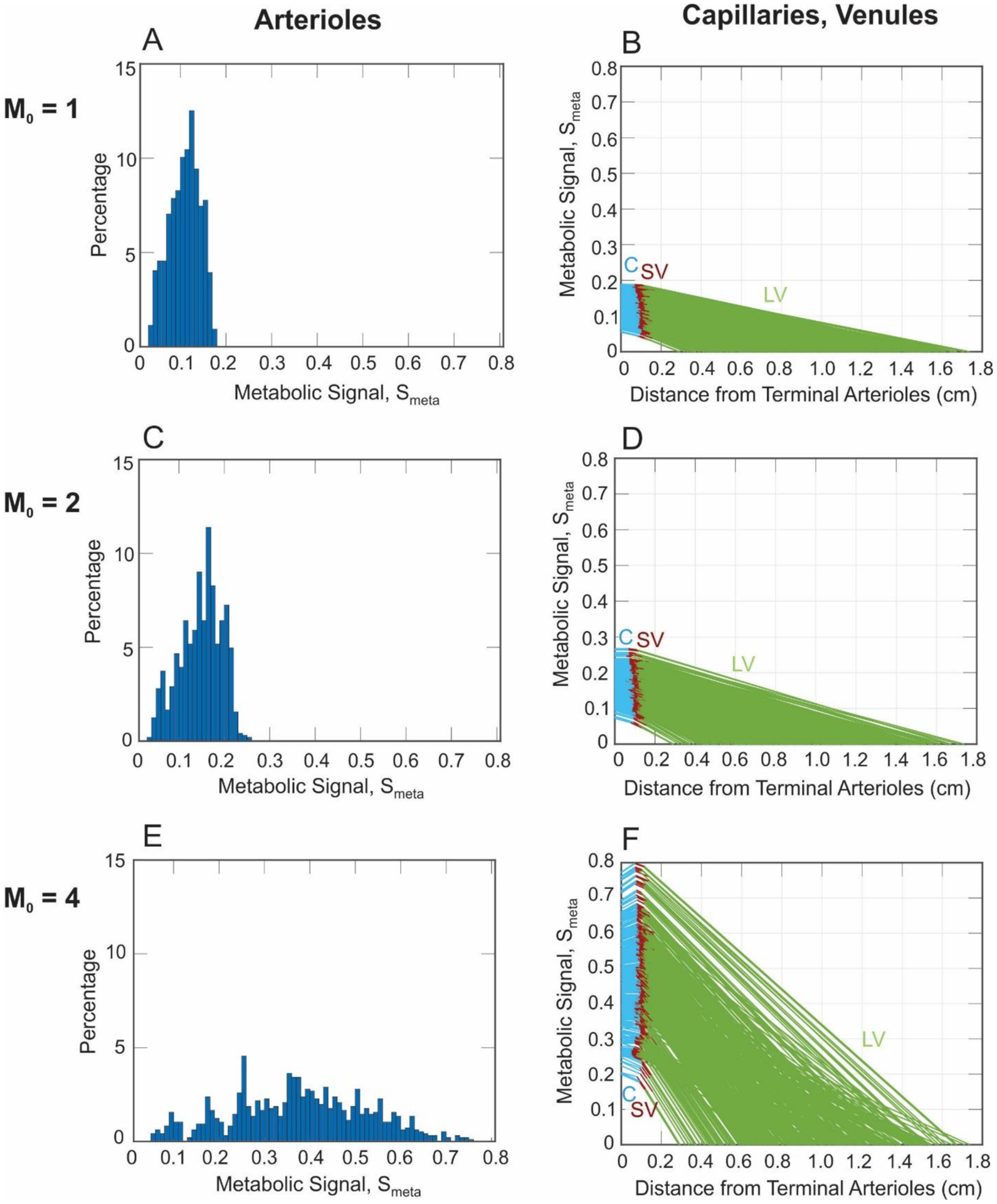
Panels (**A**,**C**,**E**): Histograms giving the percent distribution of the metabolic signal (*S*_*meta*_) in every arteriole for *M*_0_ = 1, 2, and 4 cm^3^ O_2_/100 cm^3^/min, respectively. Panels (**B**,**D**,**F**): Metabolic signal calculated at each point in the capillaries (C, blue), small venules (SV, brown), and large venules (LV, green) for *M*_0_ = 1, 2, and 4 cm^3^ O_2_/100 cm^3^/min, respectively.

Figure 5C,D shows the mean and standard deviation, respectively, of the metabolic signal (*S*_*meta*_) at the upstream end of the capillaries as oxygen demand increases. The mean and standard deviation of the signal at the downstream end of the capillaries are not shown since only minimal differences in the signal are generated along the small length of the capillaries (please see Figure 6 for signal values along each point of each pathway). The signal increases with oxygen demand (Figure 5C). As the PO_2_ in the blood approaches zero along a pathway, the local metabolic signal (*S*_*loc*_, see Equation (6)) approaches one. This explains the steep increase in both the mean and standard deviation of metabolic signals for high levels of oxygen demand. Figure 5D indicates that the model predicts not only an increase in the values of the signal but also an increase in the spread of the metabolic signal values (i.e., standard deviation) with high oxygen demand.

Figure 6 depicts the metabolic signal calculated for each pathway in the arteriolar network (Column 1) and capillary and venous compartments (Column 2). The metabolic signal is initiated in the retinal venules and is conducted upstream to the arteriolar network, where it will eventually contribute to the model’s prediction of flow regulation (future study). A minimal spread in the signal generated for *M*_0_ = 1 and *M*_0_ = 2 is observed, but a significant increase in the compartmental signal values and variability in the arteriole signal values are predicted for *M*_0_ = 4 (Figure 6E,F).

**Figure 7.**
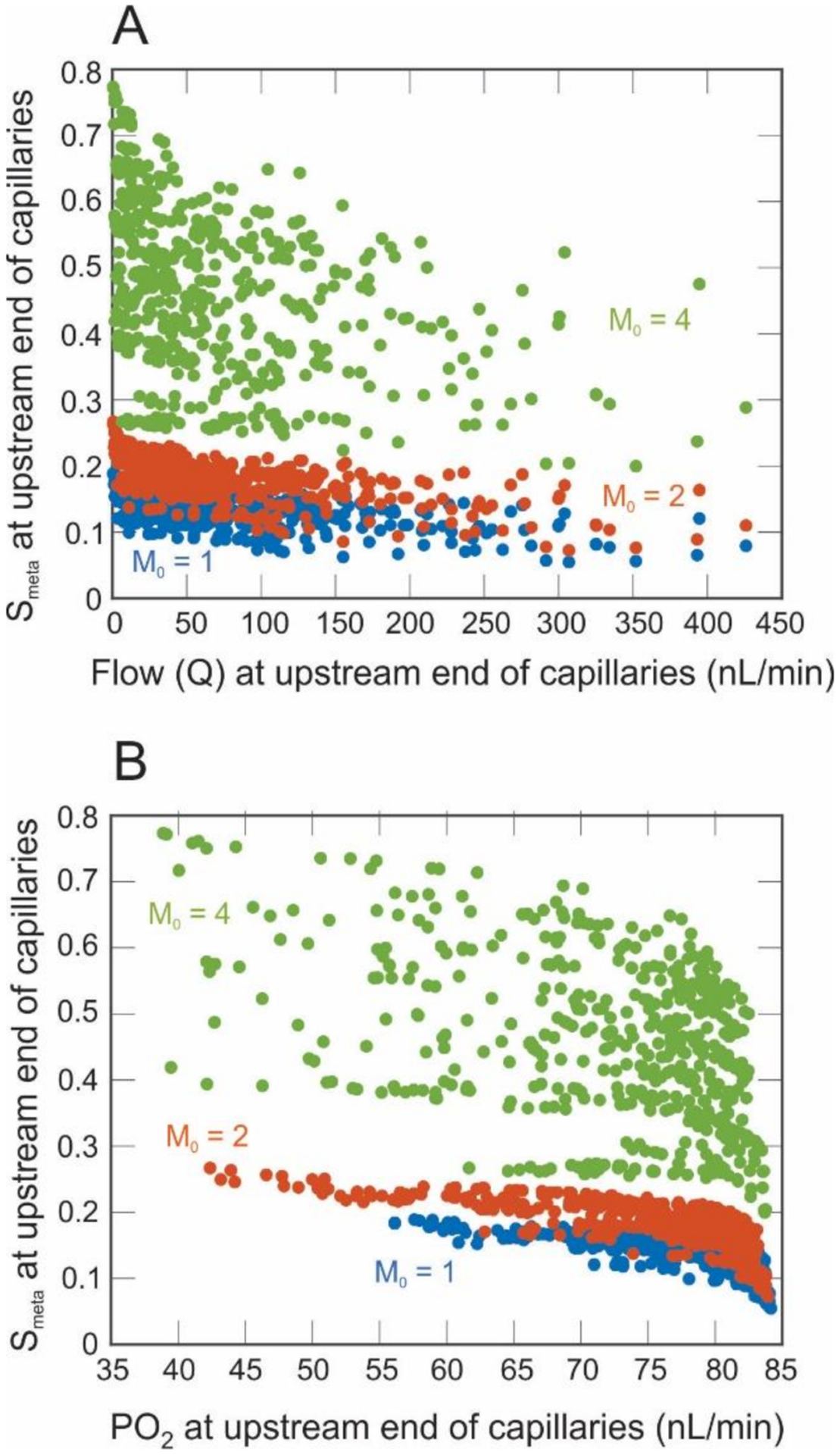
Metabolic signal (*S*_*meta*_) calculated at the upstream end of the capillaries as a function of flow (panel (**A**)) or partial pressure of oxygen (panel (**B**)) at the upstream end of the capillaries for three levels of oxygen demand: *M*_0_ = 1 (blue), 2 (red), and 4 (green) cm^3^ O_2_/100 cm^3^/min.

Figure 7A,B shows the variance in the metabolic signal reaching the arterioles as a function of flow or PO_2_ at the upstream end of the capillaries, respectively (similar results are obtained at the downstream end of the capillaries but are not shown). Pathways with the lowest flow typically have branched numerous times and contain terminal arterioles located far from the central retinal artery. These pathways tend to generate higher levels of metabolic signal, indicating a possible oxygen deficit in the network periphery. However, this general trend of increased signal with decreased flow is not very pronounced, since many pathways with high flow yield similar predictions of the metabolic signal as pathways with low flow (Figure 7A). An expected increase in metabolic signal with decreasing levels of PO_2_ is shown in Figure 7B, although several pathways generate the same level of signal for a wide range of PO_2_.

## 4. Discussion

A healthy vascular network within the retina is a critical element for preserving visual function in health and disease. The present study adapted previous models of the retinal microcirculation [2,7,25] to create a more realistic “hybrid” representation of the retinal microvasculature. An image-based murine retinal arteriolar network was translated into a heterogeneous description of a human retinal arteriolar network using scaling factors and geometric properties obtained from retinal oximetry maps. A compartmental description of the human retinal capillaries and venules [2] was connected in series to each terminal arteriole in the heterogeneous network to create a full “hybrid” description of the human retinal microcirculation. The addition of downstream vessels to the heterogeneous arteriolar network allowed for more realistic predictions of tissue and blood PO_2_, and, most importantly, allowed for the computation of metabolic signals that are conducted upstream to the arterioles.

The model predicted an expected decrease in PO_2_ (Figures 4 and 5A) throughout the human retinal microvasculature as oxygen demand (*M*_0_) was increased. In addition to a steady decrease in PO_2_ in the arterioles, a very large decrease was predicted in venular PO_2_ as *M*_0_ was increased; specifically, a decrease was predicted from mean PO_2_ values of 44 mmHg (*M*_0_ = 0.5 cm^3^ O_2_/100 cm^3^/min) to 4 mmHg (*M*_0_ = 4 cm^3^ O_2_/100 cm^3^/min). Figure 5A shows that the difference between the arteriolar and venular PO_2_ grew as the oxygen demand was increased, and Figure 5B demonstrates that the spread (as measured by the standard deviation) in PO_2_ also grew as *M*_0_ was increased. Taken together, these model predictions revealed that an increase in oxygen demand not only led to lower overall network P_O2_, but also to a larger spread in P_O2_. Thus, different regions of the retina experience vastly different oxygenation, which can lead to vastly different metabolic responses. To regulate blood flow, arterioles constrict and dilate in response to local and conducted metabolic signals. The current study demonstrated the interconnection of all vessels in an arteriolar network such that the metabolic signal (and eventual flow regulation) in a given vessel is dependent upon all other vessels in the network.

An important component of this study was the assessment of the metabolic status throughout the retina, as indicated by the metabolic responses generated in the retinal vessels. As expected, the model predicted an increase in the average metabolic signal (*S*_*meta*_) as the oxygen demand was increased (Figure 5C). However, more interestingly, the model also predicted a large increase in the *spread* of the metabolic signal throughout the network (Figures 5D and 6), similarly to the effect seen with PO_2_. While this is not surprising, given the relationship between *S*_*meta*_ and P_O2_, it is important to note that the metabolic signal varied widely throughout the network, especially for higher oxygen demand, which indicates that the metabolic signal is most sensitive at high levels of oxygen demand. As seen in Figure 6C, the value of the metabolic signal varied over an order of magnitude among the terminal arterioles of the network. This result could not be seen in a nonheterogeneous description of the retinal vasculature and demonstrates that an averaged description of the metabolic response does not adequately describe the metabolic status throughout the retina, potentially missing extreme regions at the tails of the signal distribution.

Perhaps nonintuitively, the model also revealed that vessels with equivalent values of PO_2_ exhibited differing levels of metabolic signal. For example, Figure 7B indicates an approximately threefold range in *S*_*meta*_ at the terminal arterioles (*M*_0_ = 4) despite nearly identical PO_2_ levels. This again demonstrates the importance of a whole-network approach when assessing oxygenation and metabolic status of the retina. Every vessel depends on every other vessel in the network; obtaining a measure of PO_2_ within a single vessel would not be sufficient to analyze the effect of that vessel on flow and oxygenation in the network.

This has important clinical relevance, as retinal nerve fiber tissue loss and associated visual function defects in glaucoma patients occur regionally rather than globally. Contrary to diseases such as cataract and corneal opacification that cause diffuse visual field loss, glaucomatous damage is characterized, at least in the early stages of the disease, by isolated visual field defects [29]. Importantly, glaucoma is an asymmetric disease that affects the two hemifields differently, with more pronounced damage in the superior hemifield than the inferior [30]. Little is still known about the relationship between the location of the initial visual field damage and the rate and direction of the functional disease progression, and their association with ocular and systemic factors [31]. Our comprehensive retinal model allows for regional as well as global testing which is in alignment with sectorial glaucomatous damage models and clinical presentations of the disease.

### Limitations.

The hybrid model presented here greatly improves upon retinal microcirculation models by incorporating heterogeneity within the arterioles, but it is limited by a lack of heterogeneity in the network description of the venules. Unlike the arteriolar network, which was based on confocal microscopy images, the venules were assumed to be represented as downstream fixed resistances, as in [2]. As a result, the model does not capture variation in PO_2_ or metabolic signal in the venules downstream of a given terminal arteriole, which could potentially affect the spread in signal at the upstream arterioles. However, the description of the downstream venular compartment of a given terminal arteriole *does* differ from that of another terminal arteriole in that the venular compartment parameters depend on the terminal arterioles. In this way, venular heterogeneity was represented between different pathways throughout the network. Eventually, a region-based model could extend this hybrid model to create a heterogeneous description of both the arterioles and venules for a single quadrant of the retina in hopes of obtaining even more accurate predictions of the distribution of P_O2_ and metabolic signals in the venules in a computationally tractable way.

The translation of the murine arteriolar network to the human arteriolar network also introduces some limitations, since it is not fully known if the murine network structure is identical to the human structure. Nonetheless, studies [14] indicate that the morphologies of human and murine retinas are strikingly similar and thus the murine network structure is used here as a best approximation.

Additionally, the model does not explicitly account for the intermediate and deep layers in which retinal capillaries reside. The arterioles are assumed to be located in the superficial layer of the retina, but the compartmental and Krogh model description of the capillaries do not currently account for their arrangement in three-space. Thus, future iterations of this model will establish a comprehensive multilayer heterogeneous network model geometry to improve the accuracy of model predictions when simulating metabolic requirements in the eye.

## 5. Conclusions

The current model provides predictions of metabolic signals throughout the retinal arterioles, but it does not yet predict how the arterioles change in response to these signals (i.e., blood flow regulation). Nevertheless, the hybrid model provides the necessary framework for assessing metabolic blood flow regulation in the retina and will be expanded to include blood flow regulation mechanisms as described in previous modeling work [2,17,25,32]. The ability to predict changes in arteriolar diameter and blood flow in a heterogeneous retinal microvascular network in response to changes in blood pressure or oxygen demand is critical for unraveling mechanisms involved in many ocular pathologies, especially OAG. Although experimental observations of blood flow impairment and/or biomarkers of altered retinal oxygenation in glaucoma patients have been observed for many decades, the sequential physiological events involved in such processes remain enigmatic. Our model provides a framework for predicting and interpreting hemodynamic response to physiological challenge(s) and understanding the chemical mechanisms and associated cellular responses of the retinal vasculature to stress and during disease processes. Ultimately, results of our model may allow for earlier detection and higher specificity of prognosis of ocular pathologies involving retinal hemodynamics and help identify new treatment targets for multiple eye diseases, including glaucoma.

## Figures and Tables

**Table 1.** Control state values for capillary, small venule, and large venules compartments averaged over all pathways.

Description	C	SV	LV
Diameter, *D* (μm)	6	29.5	137.3
Wall shear stress, *τ* (dyn/cm^2^)	15	15.6	14.7
Pressure Drop, Δ*P* (mmHg)	6	0.3	2.7
Number of segments, *n*	340	1	0.008
Length, *L* (cm)	0.080	0.032	0.854
Viscosity, *μ* (cP)	9.05	2.28	2.39
Flow, *Q* (cm^3^/s)	3.51×10^−9^	1.19×10^−6^	1.52×10^−4^

## References

[1] Harris A; Guidoboni G; Siesky B; Mathew S; Vercellin ACV; Rowe L; Arciero J Ocular blood flow as a clinical observation: Value, limitations and data analysis. Prog. Retin. Eye Res 2020, 78, 100841. 10.1016/j.preteyeres.2020.100841 PMC8908549 31987983

[2] Arciero J; Harris A; Siesky B; Amireskandari A; Gershuny V; Pickrell A; Guidoboni G Theoretical Analysis of Vascular Regulatory Mechanisms Contributing to Retinal Blood Flow Autoregulation. Investig. Opthalmol. Vis. Sci 2013, 54, 5584–5593. 10.1167/iovs.12-11543 PMC3747715 23847315

[3] Guidoboni G; Harris A; Cassani S; Arciero J; Siesky B; Amireskandari A; Tobe L; Egan P; Januleviciene I; Park J Intraocular Pressure, Blood Pressure, and Retinal Blood Flow Autoregulation: A Mathematical Model to Clarify Their Relationship and Clinical Relevance. Investig. Opthalmol. Vis. Sci 2014, 55, 4105–4118. 10.1167/iovs.13-13611 PMC4083771 24876284

[4] Causin P; Guidoboni G; Malgaroli F; Sacco R; Harris A Blood flow mechanics and oxygen transport and delivery in the retinal microcirculation: Multiscale mathematical modeling and numerical simulation. Biomech. Model. Mechanobiol 2015, 15, 525–542. 26232093 10.1007/s10237-015-0708-7

[5] Liu D; Wood NB; Witt N; Hughes A; Thom SA; Xu XY Computational Analysis of Oxygen Transport in the Retinal Arterial Network. Curr. Eye Res 2009, 34, 945–956. 19958111 10.3109/02713680903230079

[6] Carichino L; Harris A; Guidoboni G; Siesky BA; Pinto LA; Vandewalle E; Olafsdottir OB; Hardarson SH; Van Keer K; Stalmans I; A theoretical investigation of the increase in venous oxygen saturation levels in advanced glaucoma patients. J. Model Ophthalmol 2016, 1, 64–87.

[7] Fry BC; Coburn EB; Whiteman S; Harris A; Siesky B; Arciero J Predicting retinal tissue oxygenation using an image-based theoretical model. Math. Biosci 2018, 305, 1–9. 30149022 10.1016/j.mbs.2018.08.005

[8] Ganesan P; He S; Xu H Development of an Image-Based Network Model of Retinal Vasculature. Ann. Biomed. Eng 2010, 38, 1566–1585. 20135352 10.1007/s10439-010-9942-4

[9] Ganesan P; He S; Xu H Development of an image-based model for capillary vasculature of retina. Comput. Methods Programs Biomed 2011, 102, 35–46. 21277036 10.1016/j.cmpb.2010.12.009

[10] Popovic N; Vujosevic S; Popovic T Regional Patterns in Retinal Microvascular Network Geometry in Health and Disease. Sci. Rep 2019, 9, 16340. 31705046 10.1038/s41598-019-52659-8 PMC6841983

[11] Campbell JP; Zhang M; Hwang T; Bailey ST; Wilson DJ; Jia Y; Huang D Detailed Vascular Anatomy of the Human Retina by Projection-Resolved Optical Coherence Tomography Angiography. Sci. Rep 2017, 7, 42201. 28186181 10.1038/srep42201 PMC5301488

[12] Heegaard S; Rosenberg T; Preising M; Prause JU; Bek T An unusual retinal vascular morphology in connection with a novel AIPL1 mutation in Leber’s congenital amaurosis. Br. J. Ophthalmol 2003, 87, 980–983. 12881340 10.1136/bjo.87.8.980 PMC1771788

[13] Van Cruchten S; Vrolyk V; Lepage M-FP; Baudon M; Voute H; Schoofs S; Haruna J; Benoit-Biancamano M-O; Ruot B; Allegaert K Pre- and Postnatal Development of the Eye: A Species Comparison. Birth Defects Res 2017, 109, 1540–1567. 28941218 10.1002/bdr2.1100

[14] Smith AF; Doyeux V; Berg M; Peyrounette M; Haft-Javaherian M; LaRue A-E; Slater JH; Lauwers F; Blinder P; Tsai P; Brain Capillary Networks Across Species: A few Simple Organizational Requirements Are Sufficient to Reproduce Both Structure and Function. Front. Physiol 2019, 10, 233. 30971935 10.3389/fphys.2019.00233 PMC6444172

[15] Grunwald JE; Dupont J; Riva CE Retinal haemodynamics in patients with early diabetes mellitus. Br. J. Ophthalmol 1996, 80, 327–331. 8703884 10.1136/bjo.80.4.327 PMC505459

[16] Doblhoff-Dier V; Schmetterer L; Vilser W; Garhöfer G; Gröschl M; Leitgeb RA; Werkmeister RM Measurement of the total retinal blood flow using dual beam Fourier-domain Doppler optical coherence tomography with orthogonal detection planes. Biomed. Opt. Express 2014, 5, 630–642. 24575355 10.1364/BOE.5.000630 PMC3920891

[17] Arciero JC; Carlson BE; Secomb TW Theoretical model of metabolic blood flow regulation: Roles of ATP release by red blood cells and conducted responses. Am. J. Physiol. Circ. Physiol 2008, 295, H1562–H1571. 10.1152/ajpheart.00261.2008 PMC2593502 18689501

[18] Pries AR; Secomb TW Blood flow in microvascular networks. In Handbook of Physiology: Section 2, the Cardiovascular System, Volume IV, Microcirculation; Tuma RF, Duran WN, Ley K, Eds.; Academic Press: San Diego, CA, USA, 2008; pp. 3–36.

[19] Young D Iterative Methods for Solving Partial Difference Equations of Elliptic Type. Trans. Am. Math. Soc 1954, 76, 92–111.

[20] Ellsworth ML; Popel AS; Pittman RN Assessment and impact of heterogeneities of convective oxygen transport parameters in capillaries of striated muscle: Experimental and theoretical. Microvasc. Res 1988, 35, 341–362. 3393095 10.1016/0026-2862(88)90089-1 PMC6124310

[21] Bentley TB; Meng H; Pittman RN Temperature dependence of oxygen diffusion and consumption in mammalian striated muscle. Am. J. Physiol. Content 1993, 264, 1825–1830. 10.1152/ajpheart.1993.264.6.H1825 8322911

[22] Golub AS; Pittman RN Oxygen dependence of respiration in rat spinotrapezius muscle in situ. Am. J. Physiol. Circ. Physiol 2012, 303, H47–H56. 10.1152/ajpheart.00131.2012 PMC3404650 22523254

[23] Hsu R; Secomb TW A Green’s function method for analysis of oxygen delivery to tissue by microvascular networks. Math. Biosci 1989, 96, 61–78. 2520192 10.1016/0025-5564(89)90083-7

[24] Secomb TW; Hsu R; Park EY; Dewhirst MW Green’s function methods for analysis of oxygen delivery to tissue by microvascular networks. Ann. Biomed. Eng 2004, 32, 1519–1529. 15636112 10.1114/b:abme.0000049036.08817.44

[25] Fry BC; Harris A; Siesky B; Arciero J Blood flow regulation and oxygen transport in a heterogeneous model of the mouse retina. Math. Biosci 2020, 329, 108476. 32920096 10.1016/j.mbs.2020.108476 PMC7572810

[26] Popel A Theory of oxygen transport to tissue. Crit. Rev. Biomed. Eng 1989, 17, 257–321. 2673661 PMC5445261

[27] Krogh A The number and distribution of capillaries in muscles with calculations of the oxygen pressure head necessary for supplying the tissue. J. Physiol 1919, 52, 409–415. 16993405 10.1113/jphysiol.1919.sp001839 PMC1402716

[28] Takahashi T; Nagaoka T; Yanagida H; Saitoh T; Kamiya A; Hein T; Kuo L; Yoshida A A mathematical model for the distribution of hemodynamic parameters in the human retinal microvascular network. J. Biorheol 2009, 23, 77–86.

[29] Broadway DC Visual field testing for glaucoma—A practical guide. Community Eye Health 2012, 25, 66–70. 23520423 PMC3588129

[30] Yousefi S; Sakai H; Murata H; Fujino Y; Garway-Heath D; Weinreb R; Asaoka R Asymmetric Patterns of Visual Field Defect in Primary Open-Angle and Primary Angle-Closure Glaucoma. Investig. Opthalmol. Vis. Sci 2018, 59, 1279–1287. 10.1167/iovs.17-22980 29625450

[31] Kim JM; Kyung H; Shim SH; Azarbod P; Caprioli J Location of Initial Visual Field Defects in Glaucoma and Their Modes of Deterioration. Investig. Opthalmol. Vis. Sci 2015, 56, 7956–7962. 10.1167/iovs.15-17297 PMC4684192 26720442

[32] Carlson BE; Arciero JC; Secomb TW Theoretical model of blood flow autoregulation: Roles of myogenic, shear-dependent, and metabolic responses. Am. J. Physiol. Heart Circ. Physiol 2008, 295, H1572–H1579. 18723769 10.1152/ajpheart.00262.2008 PMC2593503

